# Supplementary material for: A novel hybrid PSO-MIDAS model and its application to the U.S. GDP forecast
Source: PLoS One. 2024 Dec 11;19(12):e0315604. doi: 10.1371/journal.pone.0315604 (PMC11633959; doi:10.1371/journal.pone.0315604)
Supplement: Attachment — Submitted filename: Response to Reviewers.docx [file pone.0315604.s002.docx]

Dear Editors and Reviewers:

Thank you for your letter and for the reviewers’ comments concerning our manuscript entitled “**A novel hybrid PSO-MIDAS model and its application to the U.S. GDP forecast**” (**ID: PONE-D-24-22830**). Those comments are all valuable and very helpful for revising and improving our paper, as well as the important guiding significance to our research. We have studied comments carefully and have made corrections which we hope meet with approval.

Reviewer #1

1. The authors need to clearly explain what makes their work new and different, especially because there are already similar PSO-MIDAS models out there. Other researchers like Xiang (2018), Rosso and colleagues (2021) have worked on models of this kind.

[1] Xiang, C. (2018). Study on Forecast of Real Estate Prosperity Index Based on MIDAS Model Corrected by Mixed Kernel Function SVM. DOI: 10.12677/AAM.2018.712182

[2] Rosso, M. M., Cucuzza, R., Di Trapani, F., & Marano, G. C. (2021). Nonpenalty machine learning constraint handling using PSO‐SVM for structural optimization. Advances in Civil Engineering, 2021(1), 6617750.

***The author‘s answer:*** We have carefully read the two papers that reviewer provided. The paper by Rosso and colleagues (2021) does not involve a PSO-MIDAS model similar to this paper. The paper by Xiang (2018) does propose a PSO-MIDAS model similar to this paper, but there is a key difference in the sample division. The paper by Xiang (2018) divides the sample into two parts: training set and test set, while our paper divides the sample into three parts: a training set, validation set and test set. The paper by Xiang (2018) optimizes the lag structure according to the forecast metrics of the MIDAS model on the training set, while our paper optimizes the lag structure according to the forecast metrics of the MIDAS model on the validation set. We find that the RMSFE of the PSO-MIDAS model with the validation set in this paper is better than that of the PSO-MIDAS model without the validation set on the U.S. GDP dataset. Since MIDAS is a time series model, the validation set is more timely than the training set, which can ensure that the PSO algorithm finds a lag structure of the MIDAS model that is more suitable for the test set.

2. The paper mainly compares its model with basic MIDAS models, but this doesn't show enough how the paper improves on what's already out there. So, the authors should also include how their model does compared to other well-known models, like those that predict things over time or models that look at data at the same frequency.

***The author‘s answer:*** There are many mixed-frequency time series prediction models, such as machine learning models such as Long Short-Term Memory (LSTM) and State-Space Models (SSM), and signal decomposition models such as Empirical Mode Decomposition (EMD). However, these models have high requirements for data scale and computing resources and are not suitable for the U.S. GDP prediction topic of this study. MIDAS is a mixed-frequency time series econometric model. The well-known ARIMA model cannot accommodate other variables except autoregressive terms and is not suitable for this study. The U-MIDAS model and ADL (ARDL) model selected in this article are both time series models that process data of different frequencies into data of the same frequency. Among them, the U-MIDAS model directly treats mixed-frequency data as data of the same frequency, and the ADL model sums high-frequency data to low-frequency frequencies. Both models are commonly used comparative models in MIDAS model research. We look forward to your recommendation on comparative models.

3. The paper needs to give a clearer and fuller explanation of how it combines the PSO algorithm with the MIDAS model, and what benefits this combination offers. The current explanation is too brief and doesn't show that the study's methods are strong and reliable.

***The author‘s answer:*** We apologize for not being able to clearly explain how the PSO algorithm is combined with the MIDAS model. We have added a new section 2.5 to explain the specific principles, processes, and advantages of the PSO-MIDAS model, and attached the pseudo-code of the PSO-MIDAS model.

4. The use of the PSO algorithm with only three explanatory variables should be justified. The authors need to clarify how the algorithm's benefits manifest in this context and why it was chosen over other variable selection methods.

***The author‘s answer:*** We are very sorry that we did not explain the role of the PSO algorithm in this study clearly. The PSO algorithm is not used as a variable selection method in this study but as an optimization algorithm for the lag orders of the MIDAS model. The lag order directly determines the number of lag variables in the model. Although only three explanatory variables were selected in this study, the introduction of the large lag order will also make the model quite complicated. As described in Section 2.1 of this article, the parameter estimation of the MIDAS model requires low-frequency data from periods 1 to $p_{Y}^{Q}$ and high-frequency data from periods 1 to $q_{X}^{M}$. This study sets the range of $p_{Y}^{Q}$ and $q_{X}^{M}$ to [1,20], [1,60]. In this interval, the selection of lag structure belongs to a nonlinear complex optimization problem, and the PSO algorithm is a suitable method to solve this problem.

5. The parameter settings listed in Table 1 need further elucidation. The authors should provide a rationale for these choices, supported by relevant references to enhance the credibility of their methodological approach.

***The author‘s answer:*** Among the PSO-related hyperparameters in this paper, the settings of Swarm size $(N_{P})$, Inertia weight $(\omega)$ and Acceleration coefficient $(c_{1}\mathrm{and}c_{1})$ are from Clerc et al. (2013). If you need more detailed hyperparameter information, you can also refer to the parameter settings in Standard Particle Swarm Optimisation by Clerc (2012). We have inserted Clerc (2012) into the references of this paper (see line 373 in the revised manuscript for details). Considering that too large a lag order will make the MIDAS model extremely complicated, we limit the range of the lag order to within 5 years, that is, 12 quarters and 60 months.

6. The manuscript relies exclusively on RMSFE to evaluate forecasting accuracy, which is overly simplistic. Commonly used metrics such as MAPE and comparative tests like Diebold-Mariano tests are missing. Incorporating these would allow for a more nuanced assessment of the model's efficacy.

***The author‘s answer:*** Considering the Reviewer’s suggestion, we have added the MAPE and Diebold–Mariano test results to the text of the article (Tables 4, 5, 7, 8, 10, 11, 13, and 14 in sections 4.1 and 4.2). The MAPE and Diebold–Mariano test results of the PSO-MIDAS model still show that the PSO algorithm improves the MIDAS model significantly, especially when forecast horizon h is large.

7. The conclusion should be expanded to include actionable policy recommendations and a discussion on the broader applications of the research. This would increase the paper's relevance and practical value.

***The author‘s answer:*** We have re-written the conclusion according to the Reviewer’s suggestion. We summarize some limitations of the PSO-MIDAS model and propose suggestions for future research of the PSO-MIDAS model (see the second paragraph of the Conclusion section for details). We hope that these suggestions can increase the practical value of the article.

Reviewer #2

1. More rigour in justifying the choice of PSO for the optimization specifically as against the general comments made in comparison with other possible techniques.

***The author‘s answer:*** We have added Section 2.5 to specifically explain the principles, processes, and advantages of the PSO-MIDAS model. In the second paragraph, we compare the PSO algorithm with well-known heuristic algorithms such as genetic algorithms and simulated annealing algorithms and explain the advantages of combining the PSO algorithm with the MIDAS model. When compared to the PSO algorithm, both of these alternatives consume more computing resources. Additionally, the genetic algorithm is better suited for solving discrete optimization problems rather than the continuous optimization problems presented in this paper. In general, the introduction of the PSO algorithm can save the researcher's calculation and time costs while ensuring the global optimum solution.

2. How does the MIDAS approach in the work of Philip H. Franses (2016) "Yet Another Look at MIDAS Regression" relate (positively or otherwise) with that adopted in this work.

***The author‘s answer:*** The MIDAS model in this paper is different from the MIDAS model in Philip H. Franses (2016) in terms of the frequency of the dependent variable. The MIDAS model in this paper has the same settings as the mainstream MIDAS model, that is, involves a dependent variable observed at a low frequency and independent variables observed at a higher frequency. However, both the independent and dependent variables of the MIDAS model in Philip H. Franses (2016) are observed at a high frequency.

3. There should be more rigour in describing and contrasting between the other Information Criterion approaches apart from the 2 mentioned (BIC & AIC) here, such as Hannan-Quim, Schwarz (or Schwarz-Bayesian) etc.

***The author‘s answer:*** Thank you for your suggestion to add information criteria. We have added the details of Hannan-Quim Criterion and Schwarz Criterion in section 2.3. Since the content of the Schwarz Criterion and BIC Criterion are the same, we have replaced the name of the BIC Criterion with the Schwarz-Bayesian Information Criterion.

4. In the analysis and conclusion, it is inferred that the accuracy of the results are enhanced with a larger forecast horizon. So how poorly does the model behave for a "small" forecast horizon and indeed, what is large enough for the forecast horizon
***The author‘s answer:*** In order to have a more comprehensive understanding of the forecast accuracy of the PSO-MIDAS model, we added the MAPE results and the Diebold–Mariano test results of the model. The Diebold–Mariano test results of the PSO-MIDAS model show that when h=3,4, the prediction accuracy of the univariate PSO-MIDAS model is significantly better than that of the MIDAS model, and when h=1,2, the prediction accuracy of the univariate PSO-MIDAS model is not significantly different from the MIDAS model. Moreover, when h=2,3,4, the forecast accuracy of the PSO-MV-MIDAS model is significantly better than the MV-MIDAS model. Through the Diebold–Mariano test, we can accurately determine the impact of the size of forecast horizon h on the forecast accuracy of the PSO-MIDAS model.

Reviewer #3

1. “When using the Mixed Data Sampling (MIDAS) regression model to forecast GDP, determining the mixed frequency lag structures using traditional lag structure selection methods reduces forecast accuracy”- Modify this line. Abstract should not start with when.

2. Author must constructively change the abstract in terms of adding only best numerical values to the result. The Author needs to write in a consistent way. Also author mention error analysis of result.

***The author‘s answer to Q1 and Q2:*** We have re-written the Abstract according to the Reviewer’s suggestion: “In this study, the traditional lag structure selection method in the Mixed Data Sampling (MIDAS) regression model for forecasting GDP was replaced with a machine learning approach using the particle swarm optimization algorithm (PSO). The introduction of PSO aimed to automatically optimize the MIDAS model's mixed-frequency lag structures, improving forecast accuracy and resolving the "forecast accuracy" and "forecast cost" weighting problem. Empirical results based on U.S. macroeconomic data show that the forecast accuracy of the univariate and multivariate PSO-MIDAS models is improved by about 10% relative to the benchmark MIDAS model, and the optimization effect is greater compared to other benchmark models. The Diebold–Mariano test results show that when the forecast horizon is large, the forecast accuracy of the PSO-MIDAS model is significantly better than other benchmark models. The innovative use of the PSO algorithm addresses the limitations of traditional lag structure selection methods and enhances the predictive potential of the MIDAS model.” We mention the average error analysis results and the Diebold–Mariano test results in the abstract.

3. Please modify the objective section for a clear understanding i.e novelty part should be clearly mentioned.

***The author‘s answer:*** According to the Reviewer’s comments, we added the innovation of the construction method of the PSO-MIDAS model and the new optimization results of the model in the Introduction (see line 110 in the revised manuscript):” This paper's main contribution lies in model construction, the machine learning algorithm is embedded in the traditional MIDAS model, and a new PSO-MIDAS model is proposed. We link the lag structure and validation set forecast metric of the MIDAS model to the particle position and fitness function of the PSO algorithm and optimize the lag structure of the MIDAS model using the PSO algorithm. According to economic and financial theory, we select the three variables of US industrial production (IP), non-farm payroll (NFP), and capacity utilization (CU) as predictors. This article compares the PSO-MIDAS model with the MIDAS model, U-MIDAS model and ADL model. The study found that the PSO-MIDAS models is improved by about 10% relative to the benchmark MIDAS model, and the optimization effect is greater compared to other benchmark models. The Diebold–Mariano test results show that when the forecast horizon is large, the forecast accuracy of the PSO-MIDAS model is significantly better than other benchmark models. This paper also extends the MIDAS model from univariate form to multivariate form. The multivariate model PSO-MV-MIDAS's forecast accuracy is still better than the multivariate benchmark models. In general, the particle swarm optimization algorithm has made a significant contribution to improving the MIDAS model's forecast accuracy.” We hope that the novelty of the article is fully interpreted.

4. Author must mention where they got data and frequency of data. Statistical analysis of data must be given in Tabular format (Like Table no 1, 10.1061/(ASCE)IR.1943-4774.0001689, not for citation purposes, only for checking Table 1 ).

***The author‘s answer:*** According to the Reviewer’s suggestion, we added Table 1 to show the descriptive statistics of the 4 variables used in this paper. As mentioned in the manuscript, the U.S. GDP data comes from the Bureau of Economic Analysis. The industrial production data and capacity utilization data come from the Federal Reserve Board, and the non-farm payroll data comes from the Bureau of Labor Statistics.

5. Please add more recent literature (2024) in terms of the PSO-MV-MIDAS Model for better understanding.

***The author‘s answer:*** We did not find any related papers using the same method as ours PSO-MV-MIDAS model, but we added two papers on the multivariate MIDAS model published in the past three years for readers' better understanding of the PSO-MV-MIDAS model (see line 78 in the revised manuscript):”Recently, some researchers begin to study the optimization of multivariate MIDAS models in price forecasting. Li et al. [18] combine the extreme learning machine and the multivariate MIDAS model to forecast natural gas price. Wang and Kang [19] combine the multivariate MIDAS model with eXtreme Gradient Boosting (XGBoost) to forecast China’s steam coal prices.”

[18]. L. Li, C. Han, S. Yao, L. Ning, Variable Weights Combination MIDAS Model Based on ELM for Natural Gas Price Forecasting, IEEE ACCESS. 2022. 10, 52075–52093.

[19]. C. Wang, W. Kang, Forecasting China’s Steam Coal Prices Using Dynamic Factors and Mixed-Frequency Data, Pol. J. Environ. Stud. 2021;30(5):4241-4254. https://doi.org/10.15244/pjoes/131856

6. Author must add study flowchart, which will clearly specify the research done in this study.

***The author‘s answer:*** We have updated the flowchart of the PSO-MIDAS model as shown in Fig 3.

7. Comparison statement (compare with other research articles) must be added in the result and discussion section to better visualize the proposed research.

***The author‘s answer:*** We did not find any articles similar to ours for comparison, but we added the MAPE metric and the Diebold–Mariano test of the univariate and multivariate PSO-MIDAS model based on the reviewers' suggestions (Tables 4, 5, 7, 8, 10, 11, 13, and 14 in sections 4.1 and 4.2). This part enriches the comparison results between the PSO-MIDAS model and the benchmark model.

8. Author must add future scope in the last portion of the manuscript.

9. Advantages and limitations of the proposed model must be added.

***The author‘s answer to Q8 and Q9:*** According to the Reviewer’s suggestion, we have re-written the conclusion part, the second paragraph of the conclusion explains some limitations of the PSO-MIDAS model and proposes suggestions for future research based on the limitations:” During the research, we also found some limitations of the PSO-MIDAS model, providing some directions for future research. First, the effectiveness of the PSO-MIDAS model is based on the assumption that the time series remains consistent in the validation set and the test set. If the potential lag structure of the target time series changes on the test set, we cannot use the validation set to calculate the potential optimal lag structure after the change. For example, our model had poor prediction results during the COVID-19 pandemic, which may be because the selected variables and their lag structures do not fully reflect the impact of the epidemic, which is also a problem faced by the benchmark MIDAS model. However, some studies have identified the inconsistency of time series through methods such as Regime Switch, which inspires the improvement of PSO-MIDAS. Second, we also tried to optimize the PSO-MIDAS lag structure through the validation set MAPE metric. Still, its model accuracy is not as good as the PSO-MIDAS model with the validation set RMSE as the fitness function. This may be because the MAPE metric is more sensitive to larger forecast errors, resulting in poor continuity of the fitness function, leading to poor PSO algorithm optimization. Therefore, the selection of forecast metrics for the fitness function is also a future research direction for PSO-MIDAS. Third, the PSO-MIDAS model can be implemented in various fields and data frequency mixtures in future studies. Including financial market variables could change the data frequency mixtures to monthly-daily or even quarterly-daily combinations. Exploring the optimization impact of the PSO-MIDAS model in different data frequency mixtures is an intriguing topic.”

10. For better analysis of the result author must add a histogram plot

***The author‘s answer:*** We added the histogram plot of the average optimization effects of the PSO-MIDAS against three benchmark models as shown in Fig 4. We also explain Figure 4 in this paragraph (see line 470 in the revised manuscript):” Finally, for better analysis of the result, we summarize the average optimization effects of the univariate and multivariate PSO-MIDAS models relative to the benchmark model into a histogram plot, as shown in Figure 4. When calculating the optimization effect of the PSO-MIDAS model, we exclude model results that are not significant in the Diebold–Mariano test. Regardless of the univariate and multivariate PSO-MIDAS models, their optimization effect relative to the MIDAS model is maintained at a stable level of 10%. It is worth noting that in multivariate models, the total lag order of the model grows exponentially, which means that the number of parameters to be estimated in the U-MV-MIDAS and MV-ADL model increases considerably, thus reducing the model’s generalisation ability. This explains why the PSO-MV-MIDAS model significantly outperforms both the U-MV-MIDAS and MV-ADL models.”
11. Author must provide a flow chart, pseudo code of proposed individual models.

***The author‘s answer:*** We have updated the flowchart of the PSO-MIDAS model as shown in Fig 3 and added a new section 2.5 to explain the specific principles, processes, and advantages of the PSO-MIDAS model, and attached the pseudo-code of the PSO-MIDAS model (see line 272 to 313 in the revised manuscript).

Thank you very much for your attention and time. Look forward to hearing from you.

Yours sincerely.

Feng Shen, Professor

shenfeng@swufe.edu.cn
