# Supplementary material for: A novel hybrid PSO-MIDAS model and its application to the U.S. GDP forecast
Source: PLoS One. 2024 Dec 11;19(12):e0315604. doi: 10.1371/journal.pone.0315604 (PMC11633959; doi:10.1371/journal.pone.0315604)
Supplement: Attachment — Submitted filename: Response to Reviewers .docx [file pone.0315604.s003.docx]

Dear Editors and Reviewers:

Thank you for your letter and for the reviewers’ comments concerning our manuscript entitled “**A novel hybrid PSO-MIDAS model and its application to the U.S. GDP forecast**” (**ID: PONE-D-24-22830R1**). Those comments are all valuable and very helpful for revising and improving our paper, as well as the important guiding significance to our research. We have studied comments carefully and have made corrections which we hope meet with approval.

Reviewer #4

1. Expand the discussion on the implications of the findings for economic policy and forecasting practices.

***The author‘s answer:*** ***:*** Considering the Reviewer’s suggestion, we have incorporated suggestions for implementing the PSO-MIDAS model in the conclusion section (see line 489 in the revised manuscript): “Based on the research findings in this article, when applying the MIDAS model for macroeconomic forecasting, it is suggested to use the PSO-MIDAS model when the forecast horizon is greater than 2, and the PSO-MV-MIDAS model when the forecast horizon is greater than 1. In terms of selecting independent variables, the forecast performance of the PSO-MIDAS model with IP and CU as independent variables is superior to that of the PSO-MIDAS model with NFP as the independent variable. Additionally, the standard deviation of the NFP variable is significantly smaller than that of other variables, so it is recommended to use independent variables with larger standard deviations”.

Thank you very much for your attention and time. Look forward to hearing from you.

Yours sincerely.

Feng Shen, Professor

shenfeng@swufe.edu.cn
