# Supplementary material for: A novel hybrid PSO-MIDAS model and its application to the U.S. GDP forecast
Source: PLoS One. 2024 Dec 11;19(12):e0315604. doi: 10.1371/journal.pone.0315604 (PMC11633959; doi:10.1371/journal.pone.0315604)
Supplement: Attachment — Submitted filename: Response to Reviewers.docx [file pone.0315604.s004.docx]

Dear Editors and Reviewers:

Thank you for your letter and for the reviewers’ comments concerning our manuscript entitled “**A novel hybrid PSO-MIDAS model and its application to the U.S. GDP forecast**” (**ID: PONE-D-24-22830R2**). Those comments are all valuable and very helpful for revising and improving our paper, as well as the important guiding significance to our research. We have studied comments carefully and have made corrections which we hope meet with approval.

Due to a change in the affiliation of the first author, Feng Shen, we updated the affiliated institute name from " Fintech Innovation Center " to " Engineering Research Center of Intelligent Finance " in line 12 of the manuscript.

Reviewer #6

1. It will be helpful to include temporal plots of actual vs forecasted GDP, and to derive conclusions about the models’ ability to forecast positive vs negative growth, since RMSE does not account for sign.

***The author‘s answer:*** Considering the Reviewer’s suggestion, we have included Figure 5 in line 495 to show the GDP forecasts of the PSO-MIDAS model with a forecast horizon of 7 months. Moreover, we have added the explanation of Figure 5 in line 486: “Additionally, we present the GDP prediction results of the PSO-MIDAS model with a forecast horizon of 7 months (the Diebold–Mariano test results show that the univariate and multivariate PSO-MIDAS models outperform the benchmark MIDAS model when forecast horizon h = 7 months), as shown in Figure 5”.

2. Line 80 - Sentence is unclear.

***The author‘s answer:*** Considering the Reviewer’s suggestion, we have rewritten this sentence to make it more clear: “Li et al. [18] integrate the extreme learning machine with the multivariate MIDAS model to predict natural gas prices.”

3. Line 98 - Chapter -> Section.

***The author‘s answer:*** Considering the Reviewer’s suggestion, we have replaced the word “Chapter” with “Section”.

4. Line 118 and Abstract - Could you please clarify the metric which is improved by 10%? The abstract mentions forecast accuracy, which could be further clarified as X-month forecast accuracy.

***The author‘s answer:*** As mentioned in line 484, when calculating the optimization effect of the PSO-MIDAS model, we exclude model results that are not significant in the Diebold–Mariano test. Regardless of the univariate and multivariate PSO-MIDAS models, their optimization effect relative to the MIDAS model is maintained at an average level of 10%. We have modified line 26, line 118 and line 486 to explain more clearly the average 10% optimization effect of the PSO-MIDAS model.

5. Eq 1 - The term u_{t+1} is undefined. It is not clear why there is a future time term on the right hand side of the equation. Same for Eq 2, 6.

***The author‘s answer:*** Considering the Reviewer’s suggestion, we have added the explanation of $\mu$ and $u_{t+1}^{Q}$ at line 140: “$\mu$ is the constant and $u_{t+1}^{Q}$ is the random error term.” Referring to Andreou et al. (2010), in the MIDAS model, the timestamp of the random error term matches that of the explained variable, so the random term $u_{t+1}^{Q}$ in Equations 2 and 6 represents a future time term $u_{t+h}^{Q}$.

[1]Elena Andreou, Eric Ghysels, Andros Kourtellos, Regression models with mixed sampling frequencies,Journal of Econometrics,Volume 158, Issue 2, 2010, Pages 246-261, ISSN 0304-4076,

6. Eq 2 - Does the equation make an assumption that the high frequency data X^{M} is monthly? This also applies to later parts of the manuscript and would be helpful to clarify.

***The author‘s answer:*** In line 132, we have reminded the reader that the time structure of this article is a monthly-quarterly structure: “Suppose we hope to forecast some low-frequency quarterly variables through mixed-frequency data in the context of monthly-quarterly data mixtures.”

7. Line 233 - It would be helpful to describe “large” as a typical number for the parameters to be estimated. For example, 100 vs 10,000.

***The author‘s answer:*** Considering the Reviewer’s suggestion, we have added an example in line 233 to help readers better understand the estimation of hyperparameters: “For example, when $p_{Y}^{Q}\mathrm{and}q_{X}^{M}\in[1,10]$, we need to estimate 100 models to find the optimal combination of $p_{Y}^{Q}$ and $q_{X}^{M}$. However, when $p_{Y}^{Q}\mathrm{and}q_{X}^{M}\in[1,100]$, the number of models required increases to 10000”.

8. Line 234 - Please expand and describe RMSFE at first usage.

***The author‘s answer:*** Considering the Reviewer’s suggestion, we have included an introduction to RMSFE in line 236.

9. Line 246 - Is the PSO algorithm capable of always finding the global optimum, or does it simply perform better than other optimization algorithms in finding the global solution? The sentence is confusing.

***The author‘s answer:*** In response to the Reviewer’s suggestion, we have rewritten this sentence for clarity: “Besides, compared to other non-random optimization algorithms, the random strategy of the PSO algorithm allows for improved global optimization capabilities”.

10. Line 265 - What is the purpose of r1 and r2 in the equation? Is this how randomness is introduced in the algorithm?

***The author‘s answer:*** The PSO algorithm uses two random variables, r1 and r2, in calculating particle velocity. This randomness is crucial, as it helps the algorithm escape from a local optimum when it gets trapped. Consequently, the random strategy employed in the PSO algorithm enhances its capability for global optimization.

11. Line 295 - It is not clear why the lag parameters are continuous. Wouldn’t they be discrete - 0, 1, 2, 3, …?

***The author‘s answer:*** The parameter optimization process of the PSO-MIDAS model is to find the parameter combination with the smallest RMSFE on the validation set. Since the goal of this optimization is to minimize the RMSFE, which is a continuous measure, we classify this as a continuous optimization problem.

12. Line 400 - The meaning of optimization effect is unclear.

***The author‘s answer:*** In Table 3 (the same as the following table), the last three columns are the ratios of the RMSFE of the PSO-MIDAS model to different benchmark models. Subtracting this ratio from 1 can give the optimization effect of the PSO-MIDAS model on the benchmark model.

13. Line 401 - The summary numbers in the text - 37%, 25%, 15% - are hard to interpret from Table 3. It would be helpful to add more explanations behind calculating these results. Also applies to later results.

***The author‘s answer:*** Considering the Reviewer’s suggestion, we have included the average ratio of the RMSFE (or MAPE) of the PSO-MIDAS model compared to various benchmark models in the last row of the table (Table 3,4,6,7,9,10,12,13). This ratio can be used to calculate the average optimization effect of the PSO-MIDAS model on different benchmark models. In addition, we added an explanation of this average ratio in line 403: “In the last row of the table, we calculated the average ratio of the RMSFE for the PSO-MIDAS model compared to three benchmark models to evaluate the average optimization performance of the PSO-MIDAS model.”

14. Line 414 - Please expand and describe MAPE at first usage.

***The author‘s answer:*** Considering the Reviewer’s suggestion, we have included an introduction to MAPE in line 422.

15. Line 419 - Please describe the Diebold and Mariano test.

***The author‘s answer:*** Considering the Reviewer’s suggestion, we have added the introduction about Diebold and Mariano test in line 429: “The Diebold-Mariano Test compares the accuracy of two forecasting methods by assessing whether the difference in forecast errors is statistically significant”.

16. Line 486 - It is subjective to state that the model was significantly better given the variability in performance. I would suggest removing “significantly”.

***The author‘s answer:*** Considering the Reviewer’s suggestion, we have removed the “significantly” in line 500. We noticed that there is also the word “significantly” in line 503, but the term "significant" here refers to its statistical meaning in the context of the Diebold–Mariano test. Therefore, we added " The Diebold–Mariano test results " to the beginning of the sentence to clarify the interpretation of "significant."

17. Line 492 - It would be helpful to include the units of forecast horizon, such as, 2 quarters.

***The author‘s answer:*** Considering the Reviewer’s suggestion, we have included the units of forecast horizon in line 506 and 507.

18. Line 491 - Is this conclusion correct for each of the three variables in the univariate model?

***The author‘s answer:*** According to the results of the Diebold–Mariano test (the “PSO-MIDAS vs. MIDAS” column of Table 5,8 and 11), when h=3,4, the univariate PSO-MIDAS model is significantly better than the MIDAS model, so the conclusion in the line 505 is applicable to each univariate model.

Thank you very much for your attention and time. Look forward to hearing from you.

Yours sincerely.

Feng Shen, Professor

shenfeng@swufe.edu.cn
